# Use of the EMPOWER brochure to deprescribe sedative-hypnotic drugs in older adults with mild cognitive impairment

**DOI:** 10.1186/s12877-017-0432-5

**Published:** 2017-01-31

**Authors:** Philippe Martin, Cara Tannenbaum

**Affiliations:** 1 Institut Universitaire de Gériatrie de Montréal, Université de Montréal, 4545 Queen Mary Road, Montreal, QC H3W 1W5 Canada; 2 Faculty of Pharmacy, University of Montreal, Quebec, Canada; 3 Faculty of Medicine, University of Montreal, Quebec, Canada

**Keywords:** Patient education, Benzodiazepines, Inappropriate prescription, Deprescribing, Discontinuation

## Abstract

**Background:**

Evidence-based mailed educational brochures about the harms of sedative-hypnotic use lead to discontinuation of chronic benzodiazepine use in older adults. It remains unknown whether patients with mild cognitive impairment (MCI) are able to understand the information in the EMPOWER brochures, and whether they achieve similar rates of benzodiazepine discontinuation.

**Methods:**

Post-hoc analysis of the EMPOWER randomized, double-blind, wait-list controlled trial that assessed the effect of a direct-to-consumer educational intervention on benzodiazepine discontinuation. 303 community-dwelling chronic users of benzodiazepine medication aged 65–95 years were recruited from general community pharmacies in the original trial, 261 (86%) of which completed the trial extension phase. All participants of the control arm received the EMPOWER brochure during the trial extension. Normal cognition (*n* = 139) or MCI (*n* = 122) was determined during baseline cognitive testing using the Montreal Cognitive Assessment questionnaire. Changes in knowledge pre- and post-intervention were assessed with a knowledge questionnaire and changes in beliefs were calculated using the Beliefs about Medicines Questionnaire. Logistic regression was used to compare knowledge gained, change in beliefs and benzodiazepine cessation rates between participants with and without MCI.

**Results:**

Complete discontinuation of benzodiazepines was achieved in 39 (32.0% [24.4,40.7]) participants with MCI and in 53 (38.1% [30.5,46.4]) with normal cognition (adjusted OR 0.79, 95% CI [0.45–1.38]). Compared to individuals with normal cognition, MCI had no effect on the acquisition of new knowledge, change in beliefs about benzodiazepines or elicitation of cognitive dissonance.

**Conclusions:**

The EMPOWER brochure is effective for reducing benzodiazepines in community-dwelling older adults with mild cognitive impairment.

**Trial registration:**

Our ClinicalTrials.gov identifier is NCT01148186, June 21^st^ 2010.

## Background

Sedative-hypnotic use is associated with cognitive impairment, and may contribute to mild neurocognitive disorders in older adults [1–3]. For this reason, both long and short-acting benzodiazepines are listed in the 2015 Beers criteria of medications to avoid in older adults [3]. A mild neurocognitive disorder is defined in the DSM-5 as a noticeable decrement in cognitive function beyond that of normal aging, which requires individuals to engage in compensatory strategies to maintain independence [4]. The term is meant to replace the previously used diagnosis of mild cognitive impairment (MCI). Over 1-in-5 community dwelling older adults have MCI at any given time, although the exact prevalence is difficult to estimate due to the variability in the criteria used, the source of subjects, the fluctuating nature of the condition and the reference standards [5, 6]. Individuals with MCI may demonstrate significant impairments in their ability to understand, reason and participate in health related decisions [7]. Longitudinal data suggest that medical decision-making capacity in patients with MCI tends to decline over time [8].

The majority of long-term benzodiazepine users aged 65 years of age and older report not being concerned about side effects, mainly because they have never been alerted to the risks [9]. However, when provided with evidence-based information about harm in the form of a mailed educational brochure, 27% of chronic users discontinued benzodiazepines within 6 months in the EMPOWER trial [10]. It remains unknown whether patients with MCI retain capacity to understand the material in the brochure, and whether they respond equally well to the educational intervention. The objective of this report is to examine whether cognitive status affected the comprehension and success rates of the EMPOWER patient-centered educational approach to the deprescribing of benzodiazepines.

## Design & methods

### Study population

Participants in the EMPOWER trial were adults aged 65 years and older with polypharmacy (≥5 medications), taking at least one chronic benzodiazepine prescription (≥3 months). Participants with self-reported epilepsy, a diagnosis of established dementia, or a mental health disorder requiring treatment with antipsychotic medication, were deemed ineligible. In order to exclude patients with undiagnosed dementia from the study, the Montreal Cognitive Assessment (MoCA) was administered at an in-home baseline screening interview. The MoCA was chosen due to its high sensitivity and specificity for distinguishing normal individuals from those with MCI [11]. Participants with a MoCA score of 26 and over were qualified as having normal cognition, while those with scores of 21 to 25 were classified as having mild cognitive impairment (MCI) [11]. Participants with scores under 21 were excluded in order to eliminate all potential cases of dementia [11]. In the original EMPOWER trial, participants randomized to the control group were wait-listed to receive the EMPOWER brochure at the end of the 6-month study period. In an extension to the trial, participants in the control arm were followed for an additional 6 months after study completion in order to evaluate their response to the EMPOWER brochure. This paper analyses all EMPOWER participants (from the intervention and control arm) having received the EMPOWER brochure and having completed the post-intervention EMPOWER assessment by 1-year (*n* = 261) [10].

### Intervention

The EMPOWER brochure consists of an 8-page paper-based benzodiazepine deprescribing tool embedded with program theories which participants received by mail. Development of the intervention has previously been described in detail [10, 12]. A generic version of the EMPOWER tool is available at http://www.criugm.qc.ca/fichier/pdf/BENZOeng.pdf. The deprescribing tool was individualized with the name of the participant’s benzodiazepine on the front page. It included true and false questions about the harms of benzodiazepines, a short paragraph describing changes in drug metabolism with age, suggestions for alternate non-drug therapies for anxiety and insomnia, a peer champion story, and a standard 21-week tapering protocol showing a picture-based diminishing schedule of full-pill, half-pill, and quarter-pill consumption. The pictogram was proposed by consumers during the development of the intervention and allows participants to apply the benzodiazepine tapering protocol regardless of the type or dose of sedative-hypnotic consumed.

### Data collection

Baseline data, including demographic characteristics and prescription details were recorded during the initial in-person interview. Follow-up data was collected by phone 1 week, 6 weeks and 6 months after each participant received the EMPOWER brochure by mail. Benzodiazepine cessation or dose reduction was ascertained using pharmacy renewal profiles, which contained information on drugs purchased, dates of purchase, dose, and quantity served. Cessation was defined as an absence of any benzodiazepine prescription renewal, sustained for a minimum of 3 months during the follow-up period. A significant dose reduction consisted of a >25% dose reduction, sustained over a minimum of 3 months when compared to baseline use. Withdrawal symptoms were measured using the benzodiazepine withdrawal symptom questionnaire 6 weeks and 6 months post-intervention. Participants reporting any withdrawal symptoms at either time point were qualified as having experienced withdrawal symptoms [13].

### Change in knowledge, beliefs and self-efficacy to taper benzodiazepines

In order to evaluate whether MCI participants understood and reacted similarly to the content of the deprescribing intervention, we measured knowledge gained, change in beliefs, improvements in self-efficacy and frequency of outreach to a healthcare professional. Change was calculated by comparing responses on the pre and post-intervention questionnaires. For knowledge, this consisted of scores on four true or false questions [12]. Beliefs about the necessity of taking benzodiazepines versus associated harms were measured by comparing the total scores on the specific section of the beliefs about medicines questionnaire [14]. Change in self-efficacy was evaluated with the Medication Reduction Self-efficacy scale [12]. Outreach to a healthcare professional was measured by self-report.

### Analysis

Participant characteristics were described using means with standard deviations for continuous data and percentages for categorical data. A chi-square test was used when comparing baseline characteristics of MCI vs non-MCI participants. Univariable logistic regression was used to determine the odds of all reported outcomes comparing participants with normal cognitive function to those with MCI. Multivariate analyses were adjusted for variables that were significantly associated with MCI at baseline, namely living arrangement, education, baseline self-efficacy and anxiety as an indication for therapy (Table 1). The results are reported as proportions with 95% confidence intervals (CI), and odds ratios (OR) with 95% CI, as appropriate. By combining participants who were randomized to the intervention, as well as the wait-list control group who received the brochure during the trial extension, the sample was powered to detect a 15% difference in proportions of individuals with and without MCI who discontinued benzodiazepines, based on an alpha of 0.05 and 80% power. The statistical significance for all analyses was set at *p* < 0.05 (two-sided). SPSS Version 20.0 (SPSS Inc. Chicago, IL, USA) was used for all analyses.

**Table 1:** Participant characteristics at baseline by cognitive status

Characteristics	All (*N* = 261)	MCI (*n* = 122)	Normal cognition (*n* = 139)	*p*-value
Female, *n* (%)	187 (71.6)	90 (73.8)	97 (69.8)	.494
Age years, Mean (SD)	74.4 (6.3)	75.3 (6.7)	73.7 (5.8)	.08
Education – college or university degree, *n* (%)	67 (25.6)	21 (17.2)	46 (33.1)	.003*
Living alone, *n* (%)	137 (52.4)	75 (61.5)	62 (44.6)	.01*
MOCA^b^, Mean score (SD)	24.5 (2.4)	23.3 (1.4)	27.4 (1.3)	.000*
General health status (poor or fair), *n* (%)	88 (32.8)	49 (32.8)	44 (31.7)	.895
Comorbidities, Mean (SD)	7.4 (2.5)	7.2 (2.4)	7.6 (2.7)	.335
Self-reported indication for benzodiazepine:				
Insomnia^c^, *n* (%)	159 (60.9)	73 (59.8)	86 (61.9)	.417
Anxiety^c^, *n* (%)	126 (48.3)	70 (57.4)	56 (40.3)	.006*
Duration of benzodiazepine use (years), Mean (SD)	10.7 (8.8)	10.3 (8.0)	10.9 (9.4)	.548
Previous attempts at cessation, *n* (%)	119 (45.6)	52 (42.6)	67 (48.2)	.321
Successful attempts, *n* (%)	41 (15.7)	14 (11.5)	27 (19.4)	.123
Benzodiazepine type^d^, *n* (%):				
Short-acting	70 (26.8)	36 (29.5)	34 (24.5)	.358
Intermediate acting	180 (70.0)	81 (66.4)	99 (71.2)	.400
Long acting	11 (4.2)	5 (4.1)	6 (4.3)	.888
Benzodiazepine Equivalent dose^a^, Mean (SD)	1.24 (.85)	1.27 (.75)	1.25 (.82)	.571
Number of medications at baseline	9.86 (3.7)	9.72 (3.8)	9.98 (3.6)	.574
Baseline Self-efficacy in tapering benzodiazepine (/100), Mean (SD)	38.1 (35.6)	31.2 (34.8)	44.1 (35.4)	.004*

*Level of significance, *p* < 0.05

^a^Benzodiazepine dose in mg of lorazepam equivalents/day

^b^MOCA: The Montreal Cognitive Assessment (scale 0–30)

^c^Based on medical diagnosis but self-reported by patients

^d^Short-acting = half-life <6 h, Intermediate acting = half-life 6–20 h, Long-acting = half-life >20 h

## Results

Participants in the post-hoc analysis consisted of older adults aged 74.4 years (6.3 year standard deviation) (Table 1). Participants were taking an average of 10 different medications and reported a mean of 7 comorbidities, with almost one third classifying their health status as unfavorable. The mean duration of benzodiazepine use was 10.7 years, indicated for insomnia and/or anxiety. Almost half (45.6%) of patients reported a previous attempt to taper their benzodiazepine. One third of the latter (15.7%) succeeded in the attempt, prior to re-initiating the drug at a later date.

One hundred twenty-two (46.7%) participants were classified as having MCI at baseline. Participants with MCI were less well educated, more likely to live alone, more likely to be taking their benzodiazepine to treat anxiety, and expressed a lower level of confidence for successful tapering than their counterparts with normal cognitive function (Table 1).

Complete discontinuation of benzodiazepines was achieved in 92 participants, with 39 (32.0% [24.4,40.7]) meeting MOCA criteria for mild cognitive impairment and 53 (38.1% [30.5,46.4]) having normal cognition (Adjusted OR = 0.79, 95% CI 0.45 to 1.38). An additional 28 participants significantly reduced their benzodiazepine dose during the same time period (12 in the normal group and 16 MCI participants). In total, 65 (46.8% [38.7–55.0]) participants with normal cognition and 55 (45.1% [36.5,53.9] MCI participants achieved dose reduction or complete discontinuation (Adjusted OR = 1.07, 95% CI 0.62 to 1.83) (Table 2).

**Table 2:** Outcomes of the EMPOWER intervention by cognitive status

Primary outcome	All (*n* = 261) (*n*, %)	MCI (*n* = 122) (*n*, %)	Normal cognition (*n* = 139) (*n*, %)	Univariable OR (95% CI)	Multivariable OR (95% CI)^a^
Cessation	92 (35.2)	39 (32.0)	53 (38.1)	0.76 [.46–1.27]	.79 [.45–1.38]
Dose Reduction	28 (10.7)	16 (13.1)	12 (8.6)	1.60 [.72–3.53]	2.04 [.86–4.83]
Cessation + Dose Reduction	120 (45.9)	55 (45.1)	65 (46.8)	.94 [.57–1.52]	1.07 [.62–1.83]
Process Outcomes					
Improvement in knowledge	157 (60.2)	75 (61.5)	82 (59.0)	1.11 [.68–1.82]	1.06 [.62–1.80]
Change in beliefs	147 (56.3)	67 (54.9)	80 (57.6)	.89 [.54–1.47]	.84 [.48–1.43]
Improved self-efficacy for tapering	144 (55.2)	68 (55.7)	76 (54.7)	1.04 [.64–1.70]	.89 [.52–1.54]
Discussed intervention with a physician	102 (39.1)	43 (35.2)	59 (42.6)	.73 [.44–1.22]	.75 [.43–1.32]
Discussed intervention with a pharmacist	55 (21.1)	26 (21.3)	29 (20.8)	1.02 [.57–1.86]	.89 [.46–1.72]
Experienced withdrawal symptoms during cessation	31 (11.9)	12 (9.8)	19 (13.7)	.69 [.32–1.49]	.60 [.27–1.35]

^a^Analyses adjusted for living arrangement, education, anxiety as an indication for benzodiazepine treatment and baseline self-efficacy

Compared to participants with normal cognition, those with MCI exhibited the same ability to acquire new knowledge and change their beliefs following the intervention. Self-efficacy to taper and experience of withdrawal symptoms was the same in both groups. Additionally, cognitive status did not affect the participants’ decision to partake in a discussion about the intervention with their healthcare provider (Table 2).

## Discussion

Although previous research indicates that individuals with MCI perform significantly worse than controls in multiple aspects of medical decision-making [7, 8, 15], we did not detect any difference in response to the EMPOWER deprescribing brochure among older adults who met MOCA criteria for MCI. Participants with MCI demonstrated improvements in knowledge and self-efficacy, were able to change their beliefs about benzodiazepines, and initiated discussions about deprescribing with a health care provider. Clinicians should be encouraged to distribute the EMPOWER brochure to their MCI patients in order to engage patients in conversations about deprescribing sedative hypnotics, leading to shared decision-making despite declining cognitive status.

### Strengths and limitations

This is the first study of its kind to explore the association between MCI and the success rates of a patient-centered educational deprescribing intervention in a community-based clinical trial of older, community-dwelling adults. As the mild neurocognitive disorder diagnosis was not yet developed at the time of the study and the MoCA’s usefulness in detecting mild neurocognitive disorder is modest [16], we categorized participants according to the older MCI diagnosis. Our results are only generalizable to patients with mild-to-moderate MCI since we used a MoCA cut-off score of 21, thus excluding the lower spectrum of MCI (19–20), which overlaps with early dementia. Additionally, as we did not re-measure scores on the MOCA at study endpoint, and were unable to ascertain whether cognition improved after discontinuation. The mean lorazepam equivalent dose was only 1.25 mg/day in both groups of participants, which may have facilitated tapering.

## Conclusions

This report illustrates that the EMPOWER brochure can be distributed to community-dwelling older adults with MCI and still work, whether directly through patient comprehension of the material or through the support of caregivers or family. The EMPOWER tool can and should be used in primary care or memory clinics for chronic benzodiazepine users who are candidates for deprescribing sedative-hypnotic medication.
